# Supplementary material for: Emotional Eating and Changes in High-Sugar Food and Drink Consumption Linked to Psychological Distress and Worries: A Cohort Study from Norway
Source: Nutrients. 2023 Feb 2;15(3):778. doi: 10.3390/nu15030778 (PMC9920951; doi:10.3390/nu15030778)
Supplement: Supplementary file 1 [file nutrients-15-00778-s001.zip › nutrients-2124695-supplementary.pdf]

Supplementary Materials

Text S1. Questionnaire items.

Item focusing on emotional eating

Q: Have you, during the past week, engaged in comfort eating or eating extra in response to feeling unhappy or unsatisfied?

A: 1(never)–2–3–4–5–6–7(everyday)

Items focusing on health-related worries

Q: I have become scared and anxious (worried) that the infection will affect myself A: Not true – somewhat true – completely true

Q: I have become scared and anxious (worried) that the infection will affect one of my closest ones

A: Not true – somewhat true – completely true

Q: I have become scared and anxious (worried) that the infection will affect elderly members of my family

A: Not true – somewhat true – completely true

Items focusing on worries regarding personal economy:

Q: I fear (am worried) that the outbreak will cause me to be laid-off temporarily or lose my job

A: Not true – somewhat true – completely true

Q: I fear (am worried) that the outbreak will lead to a poorer personal economy A: Not true – somewhat true – completely true

Items focusing on consumption of high-sugary foods and drinks

Q: How often, during the past month, did you eat or drink the following

Q1: Sugar-containing soda or soft drink

A1: Rare/never – 1-3 times/month – 1-3 times/week – 4-6 times/week – daily

Q2: Candy, cakes, biscuits or sweet desserts

A2: Rare/never – 1-3 times/month – 1-3 times/week – 4-6 times/week – daily

Items focusing on consumption of fruit and vegetables

Q: During the past month, how many portions of fruit and vegetable per day did you eat often?

A: 0-1-2-3-4-5-6-7-8-9-10 or more

Items focusing on psychological distress (Hopkins symptom checklist SCL-10)

Q: Think back to the last seven days, choose the answer that applies to you. Answer as honestly as possible.

A: ‘Not at all’, ‘A little’, ‘Quite a bit’ and ‘Extremely’

1. Suddenly scared for no reason
2. Feeling fearful
3. Faintness, dizziness or weakness

4. Feeling tense or keyed up
5. Blaming yourself for things
6. Difficulties in falling asleep or staying asleep
7. Feeling blue
8. Feelings of worthlessness
9. Feeling everything is an effort
10. Feeling hopeless about the future.

Changes in psychological distress and worries over time

In the longitudinal analysis, we treated the SCL-10 score at the first time point for every participant as constant for the sake of the linear mixed model to work. Here we assessed the changes in SCL-10 scores over time (Table S1). The cut-off for the mean SCL-10 score was ≥ 1.85, big changes were considered ≥ 0.5 and huge changes ≥ 1. Among the participants that answered the items focusing on psychological distress at the first, second and/or third time point (n= 17264), around 89% did not experienced any big changes in their SCL-10 scores over time. Moreover, only around 2% had a huge change in their SCL-10 score.

Table S1. Changes in psychological distress over time.

**SCL-10 score over time**		**Frequency**	**Percent**
**Big change**	Yes	1968	11
	No	15296	89
**Huge change**	Yes	338	2
	No	16899	98
**Total**		17264	100

We also assumed the participants' answers focusing on worries at the first time point as constant over time in the longitudinal analysis. However, here we analyzed the changes in level of worries related to health and personal economy over time. (Table S2)

Table S2. Changes in worries over time.

**Changes in worries over time**	**Frequency**	**Percent**
**Yes**	6069	34.5
**No**	11521	65.5
**Total**	17590	100

Table S3. Complete background characteristics of the participants.

Age		18-30	30-40	40-50	50-60	60-70	70>	Total
**Age distribution(%)**		3414	4228	4772	5262	4237	3055	24968
**Gender (%)**	Female	2200 (64)	2530 (60)	2768 (58)	2950 (56)	2133 (50)	1363 (45)	13944 (56)
	Male	1214 (36)	1698 (40)	2004 (42)	2312 (44)	2104 (50)	1692 (55)	11024 (44)
**BMI**	**Median**	23	25	25	26	26	25	25
	**25% -75 %**	21-26	22-28	23-28	24-29	23-28	23-28	23-28
**BMI Categories (%)**	Underweight	114(4)	58(1)	31 (1)	36 (1)	38 (1)	32 (1)	309 (1)
	Normal	2038 (64)	1285 (55)	1500 (48)	525 (42)	43 (39)	3 (21)	3498 (49)
	Overweight	739 (23)	1278 (32)	1673 (37)	2103 (43)	1717 (43)	1154 (41)	8664 (37)
	Obese	298 (9)	516 (13)	704 (16)	808 (16)	553 (14)	285 (10)	3164 (14)
**Number of household members >18 y/o**	Living alone	503 (15)	813 (19)	885 (19)	1027 (20)	1193 (28)	1234 (40)	5655 (23)
	living with at least one person	2911 (85)	3415 (81)	3887 (81)	4235 (80)	3044 (72)	1821 (60)	19313 (77)
**Living with own children (<18 y/o)**		811 (24)	2595 (61)	3486 (73)	1565 (30)	334 (8)	247 (8)	9038 (36)
**Education level**	Primary school	414 (13)	150 (4)	182 (4)	272 (5)	358 (9)	375 (13)	1751 (7)
	High school/ trade school	1177 (36)	773 (19)	932 (20)	1604 (31)	1346 (32)	959 (32)	6791 (28)
	≤3 years of higher education	866 (26)	1046 (26)	1098 (24)	1243 (24)	938 (22)	692 (23)	5883 (24)
	≥ 4 years of higher education	828 (25)	2109 (52)	2412 (52)	2022 (39)	1531 (37)	969 (32)	9871 (41)
**Employment prior to COVID-19 (%)**	Employed (full/part time)	2178 (64)	3548 (84)	4175 (87)	4550 (86)	2415 (57)	201 (7)	17067 (68)
	Student	1578 (46)	251 (6)	101 (2)	31 (1)	6 (0)	3 (00	1970 (8)
**Household income (%)**	Low	1041 (36)	522 (13)	462 (10)	364 (8)	234 (6)	314 (13)	2937 (13)
	Medium	1098 (38)	1944 (49)	2259 (51)	1800 (38)	1262 (35)	1204 (50)	9567 (44)
	High	734 (26)	1464 (37)	1678 (38)	2536 (54)	2109 (59)	886 (37)	9407 (43)
**COVID consequences in the initial period (%)**	Temporarily laid-off	505 (15)	394 (9)	358 (8)	411 (8)	216 (5)	18 (1)	1902 (8)
	Lost employment	76 (2)	60 (1)	38 (1)	39 (1)	23 (1)	5 (0)	241 (1)
	Home office	767 (21)	696 (16)	718 (15)	738 (14)	630 (15)	507 (17)	4056 (16)
	Placed in quarantine	712 (21)	2069 (49)	2558 (54)	2477 (47)	1243 (29)	70 (2)	9129 (37)
**Substantial worry (%)**	Related to personal economy	998 (29)	514 (22)	561 (17)	207 (16)	12 (10)	1 (7)	1385 (19)
	Health-related	1889 (55)	2089 (490	2181 (46)	2507 (48)	1559 (37)	887 (29)	11112 (45)
**Psychological distress (%)**		1403 (41)	1211 (29)	935 (20)	823 (16)	475 (11)	247 (8)	5094 (20)

Table S4. Degree of emotional eating and associations to psychological distress, worries and baseline characteristics (linear mixed model presenting absolute coefficients with 0 indicating no difference/change).

Estimates presented as coefficients, with their 99% confidence intervals		
	Fixed effects	Time trend (per year)
**Age**		
18-29	0 (reference)	0 (reference)
30-39	0.048 (0.035;0.061)	-0.015 (-0.027;-0.003)
40-49	0.033 (0.020;0.046)	-0.015 (-0.026;-0.003)
50-59	0.024 (0.012;0.036)	-0.007 (-0.018;0.004)
60-69	-0.001(-0.014;0.012)	-0.002 (-0.014;0.009)
70+	-0.031(-0.045;-0.016)	0.014 (0.001;0.026)
**Sex**		
Male	0 (reference)	0 (reference)
Female	0.055 (0.048;0.062)	-0.021 (-0.026; -0.015)
**Living with other adult(s)**		
No	0 (reference)	0 (reference)
Yes	-0.02 (-0.03; -0.01)	0.001 (-0.002; 0.021)
**Living with own children (<18 years of age)**		
No	0 (reference)	0 (reference)
Yes	0.000 (-0.009; 0.008)	0.040 (0.032; 0.048)
**Health-related worries**		
None or some	0 (reference)	0 (reference)
Substantial	0.018 (0.011;0.025)	-0.009 (-0.015;-0.002)
**Worries related to economy**		
None or some	0 (reference)	0 (reference)
Substantial	0.044 (0.035;0.054)	-0.001 (-0.011;0.010)
**Psychological distress** (0=no to 1=extreme)	0.63 (0.61;0.65)	-0.18 (-0.20;-0.17)

*Baseline constant of emotional eating: 0.06 (0.05;0.07), time trend: -0.07 (-0.08;-0.05)

Table S5. High-sugar food and drink intake and associations to psychological distress, worries and base-line characteristics (linear mixed model presenting absolute coefficients with 0 indicating no difference/change).

Estimates presented as coefficients, with their 99% confidence intervals		
	Fixed effects	Time trend (per year)
**Age**		
18-29	0 (reference)	0 (reference)
30-39	-0.033 (-0.046;-0.021)	0.001 (-0.008;0.010)
40-49	-0.062 (-0.074;-0.050)	-0.003 (-0.012;0.006)
50-59	-0.105 (-0.117;-0.093)	-0.004 (-0.013;0.004)
60-69	-0.139 (-0.151;-0.127)	-0.003 (-0.011;0.006)
70+	-0.151(-0.165;-0.138)	0.004 (-0.005;0.014)
**Sex**		
Male	0 (reference)	0 (reference)
Female	-0.020 (-0.026;-0.013)	-0.009 (-0.013;-0.005)
**Living with other adult(s)**		
No	0 (reference)	0 (reference)
Yes	0.011 (0.004;0.018)	-0.003 (-0.012;0.006)
**Living with own children (<18 y/o)**		
No	0 (reference)	0 (reference)
Yes	0.029 (0.021;0.036)	-0.038 (-0.044;-0.032)
**Health-related worries**		
None or some	0 (reference)	0 (reference)
Substantial	0.004 (-0.001;0.010)	0.001 (-0.004; 0.006)
**Worries related to personal economy**		
None or some	0 (reference)	0 (reference)
Substantial	0.008 (0.000;0.016)	0.004 (-0.004;0.012)
**Psychological distress** (0=no to 1=extreme)	0.11 (0.09;0.13)	-0.014 (-0.029; 0.000)

* Baseline constant of High-sugar food and drink intake: 0.38 (0.36;0.39), time trend: 0.034 (0.022;0.045)

Table S6. Servings per day of fruits and vegetables and associations to psychological distress, worries and baseline characteristics (linear mixed model presenting absolute coefficients with 0 indicating no difference/change).

Estimates presented as coefficients, with their 99% confidence intervals		
	Constant/fixed effects	Time trend (per year)
**Age**		
18-30	0 (reference)	0 (reference)
30-40	0.006 (-0.008;0.019)	0.007 (-0.005;0.020)
40-50	-0.007 (-0.021;0.007)	0.009 (-0.004;0.022)
50-60	0.010 (-0.003;0.023)	0.006 (-0.006;0.018)
60-70	0.030 (0.017;0.044)	0.004 (-0.009;0.016)
70+	0.049 (0.034;0.064)	-0.0005 (-0.014;0.013)
**Sex**		
Male	0 (reference)	0 (reference)
Female	0.045 (0.038;0.052)	-0.002 (-0.009;0.004)
**Living with other adult(s)**		
No	0 (reference)	0 (reference)
Yes	0.008 (-0.001;0.017)	-0.001 (-0.015;0.012)
**Living with own children (<18years)**		
No	0 (reference)	0 (reference)
Yes	0.001 (-0.008;0.010)	-0.005 (-0.014;0.004)
**Health-related worries**		
None or some	0 (reference)	0 (reference)
Substantial	-0.001 (-0.008;0.006)	0.002 (-0.005;0.009)
**Worries related to personal economy**		
None or some	0 (reference)	0 (reference)
Substantial	0.011 (0.001;0.021)	-0.005 (-0.017;0.006)
**Psychological distress** (0=no to 1=extreme)	-0.040 (-0.063;-0.016)	0.022 (0.001;0.043)

* Baseline constant of fruit and vegetables intake: 0.25 (0.23;0.26), time trend: -0.002 (-0.019;0.014)

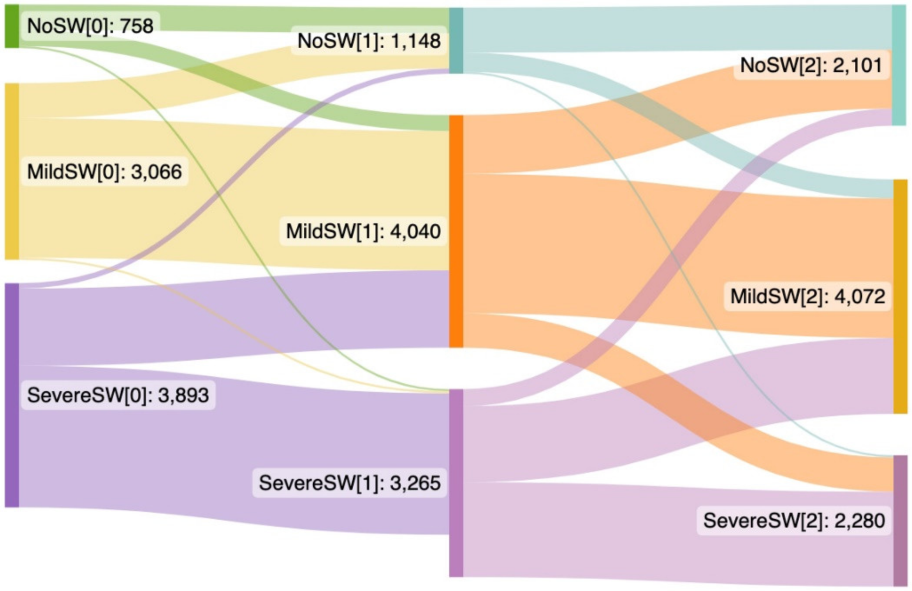

Figure S1. Sankey plot: Substantial worries over time *.

* This plot includes the participants that answered the items focusing on worries related to personal economy and health on the questionnaire at the first time point (t_0), second time point (t_1) and third time point (t_2) (n=8453). Those participants that reported no health worries and no worries related to personal economy were considered in the no substantial worry group (NoSW), those who had some worries regarding health or economy were considered in mild substantial worry group (MildSW), and those who had either high levels of worries related to health or economy were placed in severe substantial worry group (SevereSW). As it is shown in the plot, changes in worries from SevereSW to NoSW and the other way is quite few. Some fluctuations is seen between time points, especially from SevereSW to MildSW; but, the overall numbers of the groups did not change considerably.
